# Experiments on Digestion

**Published:** 1859-03

**Authors:** 


*Experiments on Digestion.*—Dr. Geo. Harley read a communication on this subject before the Section of Physiology of the British Association for the Advancement of Science, at its late meeting in Leeds.

The communication was illustrated by numerous experiments showing the properties of the saliva, the gastric juice, the bile, and the pancreatic secretion. The author stated that, contrary to an opinion lately published by Bernard, he had found that the human saliva contains both sulphocyanide of potassium and iron. The latter substance, however, can only be detected after the organic matters contained in the secretion are destroyed by burning. Dr. Harley had ascertained that a person of nine stone secreted between one and two pounds of saliva in twenty-four hours. The gastric juice, the author said does not destroy the power possessed by the saliva of transforming starch into sugar; consequently, the digestion of amylaceous food is continued in the stomach. The gastric juice has the property of changing cane into grape sugar. The author made some remarks about the cause of the gastric juice not digesting the living stomach; and said that his experiments showed that it is not the epithelium lining the organ which prevents its being digested, but the layer of thick mucus which covers its walls. When the latter substance is absent, the gastric juice attacks the wall of the living stomach, and digests them, causing perforation and death. As regards the bile, it seems that this secretion takes an active part in rendering the fatty matters of our food capable of being absorbed into the system. The most curious of all the digestive fluids, however, is the pancreatic secretion, for it unites in itself the properties of all the others. It not only transforms starch and other such substances into sugar, but it emulsionizes fats, and even digests protein compounds. As a remedy in indigestion, pancreatine should be greatly superior to pepsine, which can only digest one kind of food, namely, protein. The author said he had been laboring to obtain pancreatine in a perfectly pure state, and had been to a certain degree successful. With pancreatine, we should be able to digest any kind of food we pleased; and, therefore, the obtaining of it in a sure state of purity would be an invaluable boon to suffering humanity.—*British Med. Journal,* from *Amer. Journal Med. Sciences.*

